# Facilitated patient experience feedback can improve nursing care: a pilot study for a phase III cluster randomised controlled trial

**DOI:** 10.1186/1472-6963-13-259

**Published:** 2013-07-04

**Authors:** Rachel Reeves, Elizabeth West, David Barron

**Affiliations:** 1 School of Health and Social Care, University of Greenwich, London SE9 2UG, UK; 2 Saïd Business School, University of Oxford, Oxford, OX1 1HP, UK

**Keywords:** Patient surveys, Patient experience, Patient satisfaction, Hospitals, Inpatients, Quality improvement, Patient-centred care

## Abstract

**Background:**

England’s extensive NHS patient survey programme has not fulfilled government promises of widespread improvements in patients’ experiences, and media reports of poor nursing care in NHS hospitals are increasingly common. Impediments to the surveys’ impact on the quality of nursing care may include: the fact that they are not ward-specific, so nurses claim “that doesn’t happen on my ward”; nurses’ scepticism about the relevance of patient feedback to their practice; and lack of prompt communication of results. The surveys’ impact could be increased by: conducting ward-specific surveys; returning results to ward staff more quickly; including patients’ written comments in reports; and offering nurses an opportunity to discuss the feedback. Very few randomised trials have been conducted to test the effectiveness of patient feedback on quality improvement and there have been few, if any, published trials of ward-specific patient surveys.

**Methods:**

Over two years, postal surveys of recent inpatients were conducted at four-monthly intervals in 18 wards in two NHS Trusts in England. Wards were randomly allocated to *Basic Feedback* (ward-specific printed patient survey results including patients’ written comments sent to nurses by letter); *Feedback Plus* (in addition to printed results, ward meetings to discuss results and plan improvements) or *Control* (no active feedback of survey results). Patient survey responses to questions about nursing care were used to compute wards’ average Nursing Care Scores at each interval. Nurses’ reactions to the patient feedback were recorded.

**Results:**

Conducting ward-level surveys and delivering ward-specific results was feasible. Ward meetings were effective for engaging nurses and challenging scepticism and patients’ written comments stimulated interest. 4,236 (47%) patients returned questionnaires. *Nursing Care Scores* improved more for *Feedback Plus* than *Basic Feedback* or *Control* (difference between *Control* and *Feedback Plus* = 8.28 ± 7.2 (*p* = 0.02)).

**Conclusions:**

This study provides preliminary evidence that facilitated patient feedback can improve patients’ experiences such that a full trial is justified. These findings suggest that merely informing nurses of patient survey results in writing does not stimulate improvements, even if results are disaggregated by ward, but the addition of ward meetings had an important and significant impact.

## Background

One of the assumptions underlying England’s National Health Service (NHS) policy is that giving feedback about patients’ experiences to healthcare organisations will drive improvements [1-5]. Specifically, in 2000, the *NHS Plan* pledged that a patient survey would “secure year-on-year improvements in patient satisfaction”. Since 2002, the Inpatient Survey for Acute NHS Trusts has been conducted in all acute NHS hospitals in England, sampling approximately 135,000 adult patients each year. The questionnaires, sent by post, cover waiting times, doctors’ and nurses’ attitudes, staff responsiveness, hospital food and cleanliness, patient information, co-ordination of care and patients’ dignity [6]. To date, more than 700,000 questionnaires have been returned.

Over the last 10 years, the surveys have detected improvements in the national results, but the aspects of care that have improved are those that can be linked to national targets or high-profile media campaigns (such as waiting times for inpatient admissions and ward cleanliness). The results for most questions have stayed the same or, in some cases, have deteriorated. For example, in 2011, only 53% of patients said their call bells were usually answered within two minutes (10% worse than 2004) and 21% said nurses “talked in front of them as if they were not there” (3% worse than 2002) [7,8]. There is little evidence that the survey programme itself has driven any improvements in patients’ experiences.

Qualitative research suggests that NHS staff recognise the national surveys’ methodological robustness [9,10] but that there are a number of barriers to the surveys’ impact. Conducting trust-level surveys means that members of staff do not recognise the results as their own, often claiming “that doesn’t happen on my ward” [9,11-13]. Currently, the survey results are communicated to senior hospital managers; they are not communicated *directly* to those who are towards the bottom of hospitals’ hierarchies, even though they are the staff members who are disproportionately responsible for making day-to-day decisions about the way care is delivered [14]. The extent to which managers in many NHS trusts successfully “cascade” results to clinical staff is acknowledged to be inadequate [9]. Other barriers to the impact of patient feedback include poor understanding of survey methods and statistics; scepticism of clinical staff about the relevance of survey data to their practice; and delays between data collection and feedback, so staff may argue that circumstances have changed and care has now improved [9,11-13]. Clinicians’ engagement with patient feedback data may be enhanced by including patients’ comments alongside numerical data [9,15,16].

Arguably, the surveys’ lack of impact could also be attributed to an over-emphasis on data collection *per se*, rather than using results to improve the quality of care. The recent promotion of “real-time” feedback will increase the volume of survey data collected by NHS trusts while reducing the rigour of survey methods [5]. Uniform data collection methods are not required to conduct the “Friends and Family” survey, which covers all NHS inpatients and emergency department attendees from April 2013 onwards, and there are no formal mechanisms in place to ensure that the survey data are used for quality improvement [17].

Very few randomised controlled trials have been conducted to test the effects of quality improvement programmes [18]. The overall aim of this research programme is to refine and test an intervention intended to overcome barriers to the impact of patient feedback on clinicians’ behaviour. Nurses were selected as the target group of clinicians because many of the survey’s questions are about aspects of care that are provided by nurses; poor nursing care has been the focus of many recent media reports about negative patient experiences; [19] and nurses tend to work in coherent units (wards), which can be linked to specific groups of patients treated on those wards. The intervention was designed to: (1) improve nurses’ willingness to accept ownership of the patient feedback by making survey results ward-specific; (2) increase the immediacy of the feedback by shortening the time taken to return survey results to approximately twelve weeks after patients’ discharges (compared to approximately nine months to return national patient survey results to NHS trust managers); and (3) engage nurses’ interest by including patients’ comments alongside numerical results in printed reports. In addition, an enhanced version of the intervention included ward meetings to facilitate nurses’ engagement with the feedback; counteract scepticism about the relevance of the feedback to their practice; support them to act on the findings and give them an opportunity to ask questions about the surveys’ methodological reliability and validity.

To enhance the intervention’s acceptability to policy makers and NHS managers, and to maximise its chances of being adopted throughout England’s NHS hospitals, the Care Quality Commission’s (CQC’s) standard questionnaire and survey method currently used for inpatient national survey programme were used to obtain patient feedback [20]. The pre-tested questionnaire and postal survey method conform to widely-accepted methodological standards [21]: centrally monitored protocols ensure that participating organisations comply with a uniform sampling method and postal survey; questionnaires are tested to ensure that they cover patients’ priorities and can be understood by different demographic groups [22,23]. Questions are purposely designed to facilitate quality improvements by providing actionable feedback to healthcare professionals: patients are asked to report *what happened* to them regarding specific aspects of their care episode, rather than asking them to rate their satisfaction more generally [24]. The sensitivity of the inpatient survey to changes in patients’ experiences is supported by the changes in the national results noted above.

This was a pilot study, designed to guide the planning for a large-scale trial corresponding to phase III of the Medical Research Council (MRC) guidelines on developing and evaluating complex interventions [25-27]. The main aims of the pilot were: to test the feasibility of conducting ward-level surveys, providing ward-level data and conducting ward meetings; to provide preliminary evidence on the effectiveness of two levels of the intervention; and to provide an estimate of the number of wards that would constitute the sample size needed for a definitive trial. For the purposes of measuring patients’ experiences at different time points during the study, patients were surveyed in ward clusters and average composite *Nursing Care Scores* were computed at each interval using responses to nursing questions from different groups of patients who were recently discharged at each survey interval.

## Methods

### Ethical review

Ethical approval for the study was obtained from Bexley & Greenwich Research Ethics Committee (reference 08/H0809/55).

### Setting

The study was conducted in two single-hospital-site Acute Hospital NHS Trusts in London (Trust A and Trust B). The data collection took two years from 2009 to 2010 in a total of 18 wards.

### Design

Adult non-maternity wards that treated inpatients wards eligible for inclusion. There were 18 eligible wards in Trust A and 14 in Trust B. Two levels of the intervention were tested: *Basic Feedback* and *Feedback Plus*. The participating wards were randomly allocated in equal numbers to *Control*, *Basic Feedback* (on which nurses would receive written feedback only), and *Feedback Plus* (where, in addition to written feedback, ward meetings were held). Separately for each trust, all eligible wards were listed in one column of an Excel spreadsheet, then using the “Rand()” function, a random number was generated alongside each ward. The ward list was then sorted by the random number and the first three wards were assigned to the *Basic Feedback* condition*,* the next three to *Feedback Plus* and the next three to *Control.* Once nine wards were selected for each trust, the remaining wards were not included in the study. All ward managers, matrons and Directors of Nursing were aware throughout the study of the condition to which wards had been randomised.

### Patient sampling criteria

Each ward’s sample comprised de-duplicated consecutively-discharged patients from the previous two months, up to a maximum of 160 patients per ward. All adult patients were eligible for inclusion in the sample. At the end of each four-month survey interval, NHS trust record-keeping departments were asked to collate the sample of patients using sampling instructions adapted from those used for the national Inpatient Survey to allow the addition of ward information. The information about each sampled patient included admission and discharge dates, year of birth, sex, and ward on which patients had spent the longest time. Patients who had spent time on more than one ward were assigned to the sample for the ward on which they had spent the most time. If a ward’s sample included more than 160 patients, the most recent 160 were included and the remainder were removed from the sample.

### Patient survey method

At four-monthly intervals, patient feedback was gathered using the CQC’s Inpatient Questionnaire (2009/2010 version) and postal survey method. Prior to mailings, patients known to have died were removed from the sample. Researchers did not have access to patients’ name and address information. The CQC-approved survey company carried out the mailings under a similar contractual arrangement with the NHS trust as used for the national patient surveys. For the purposes of carrying out mailings, a separate file included the sampled patients’ names and addresses and code numbers corresponding to the anonymous patient sample file. The contractors sent out code-numbered postal surveys to patients, monitored whether or not they had responded and sent out reminders accordingly.

Questionnaires were sent out by post to patients’ home addresses. Letters accompanying questionnaires corresponded to the standard covering letter used for the CQC Inpatient Survey, with the added request that patients should relate their responses to the ward named in the covering letter. Consistent with the CQC survey protocol, the covering letter included details of a free telephone number, by which patients or their relatives could speak to a member of the survey contractor’s staff if they had any questions about the survey. Included with the questionnaire was a free postage envelope in which to return the questionnaire. At two-weekly intervals, two postal reminders were sent to non-responders. The second reminder included a duplicate questionnaire.

### Intervention

#### *Written feedback*

Individual letters sent to nurses employed on *Basic Feedback* and *Feedback Plus* wards and their matrons included detailed ward-level patient survey results. The printed survey results comprised: (1) a bar chart for each of 10 to 15 questions about nursing care comparing the six feedback wards in one hospital (with the target’s own ward highlighted in a different colour) and the NHS average. The bars showed the percentage of positive responses to a question; (2) as the study progressed and historical data were available, 10 to 15 line graphs, each showing the ward’s changes over time on one question about nursing care; and (3) a transcription of the comments patients had written in the spaces at the end of the questionnaire, under the headings “Was there anything particularly good about your care?”; “Was there anything that could be improved?” and “Any other comments?”. A covering letter to the nurse explained the purpose of the study and included contact details for the lead researcher.

#### *Ward meetings*

On *Feedback Plus* wards, the printed survey results were supplemented with ward meetings with the researchers, during which the results discussed and, if necessary, explained, and there was an opportunity to ask questions about survey methods and to plan improvements in practice. Ward managers were responsible for inviting as many or few other members of their nursing team as they chose. Meetings took place in ward offices within normal working hours. At the beginning of each meeting, nurses’ consent to take part in the study was obtained with the understanding that any comments they might make would not be attributed to individual nurses, and any details identifying individuals or their ward affiliation would be removed before they were shared with nurse managers or anyone else. Following meetings, the written survey results were sent individually to all of the ward’s nurses (regardless of whether they had attended the meeting) in the same format as used for *Basic Feedback*.

*Control* wards closely matched the usual condition for the annual national surveys. Their data were included in the set of results given to the Director of Nursing but no special efforts were made to communicate them at ward level. However, results would be made available to ward nurses if they requested them.

### Feasibility objectives

The intervention’s feasibility was assessed according to the following criteria:

•Could NHS trust record-keeping staff collate accurate ward-specific samples of patients?

•Could ward-level postal surveys be conducted successfully?

•Could researchers use the data from the returned questionnaires to produce ward-specific reports on the survey results?

•Would the researchers successfully arrange and conduct meetings on *Feedback Plus* wards and would nurses attend them?

•Would ward nursing staff understand the ward-specific survey reports and show an interest in them?

•Would nurses engage with the patient feedback and accept it as valid information about the quality of care they had provided?

### Feasibility data

The data used to assess feasibility were collected during the course of the study. The survey contractor was asked to compare their experience of carrying out the national inpatient surveys and study’s surveys, and to report any differences. They were also asked to note the volume of calls to the telephone helpline, and to record the subjects of the telephone calls. The researchers made notes about nurses’ comments in ward meetings and conducted a telephone survey of ward managers on *Basic Feedback* wards, asking them what, if any, actions they had implemented as a result of the patient feedback. The qualitative data collected in meetings and telephone calls was examined for common themes.

### Outcome measure

The aim of the intervention was to improve nursing care in general, rather than to focus on specific aspects of nursing care. Therefore, the mean of a subset of 20 questions was used to derive a composite *Nursing Care Score*. The selected questions, listed below, were those which were most closely associated with the quality of ward nursing. Each question was scored between 0 and 100, where higher scores indicate better care. For the responders in this study, *Nursing Care Scores* ranged between 5 and 100 and the mean score was 75.4.

Survey questions included in the Nursing Care Score

1. Were you ever bothered by noise at night from other patients?

2. Were you ever bothered by noise at night from staff?

3. In your opinion, how clean was the room or ward that you were in?

4. How clean were the toilets and bathrooms that you used in hospital?

5. Did you get enough help from staff to eat your meals?

6. When you had important questions to ask a nurse, did you get answers that you could understand?

7. Did you have confidence and trust in the nurses treating you?

8. Did nurses talk in front of you as if you weren’t there?

9. As far as you know, did nurses wash or clean their hands between touching patients?

10. Did you find someone on the hospital staff to talk to about your worries and fears?

11. Do you think the hospital staff did everything they could to help control your pain?

12. How many minutes after you used the call button did it usually take before you got the help you needed?

13. Was your discharge delayed by waiting for doctors or medicines and if so how long was the extra wait?

14. Before you left hospital, were you given any written or printed information about what you should or should not do after leaving hospital?

15. Were you told how to take your medication in a way you could understand?

16. Did a member of staff tell you about any danger signals you should watch for after you went home?

17. Did hospital staff tell you who to contact if you were worried about your condition or treatment after you left hospital?

18. Overall, did you feel you were treated with respect and dignity while you were in the hospital?

19. Overall, how would you rate the care you received?

20. Did you want to complain about the care you received in hospital?

### Statistical analysis plan

The outcome measure described above was designed to be analysed using a standard model that allows examination of its variation over the six time periods using a form of multilevel regression. The statistical model to be estimated is shown in equation (1):

(1) $$\begin{array}{c} Y_{\mathrm{wt}}=\beta_{0w}+\beta_{1}N_{w}+\beta_{2}G_{\mathrm{bw}}+\beta_{3}G_{\mathrm{pw}}+\beta_{4}t+\beta_{5}G_{\mathrm{bw}}t\\ \;+\beta_{6}G_{\mathrm{pw}}t+\epsilon_{\mathrm{wt}}\\ \beta_{0w}=\gamma_{0}+\delta_{w} \end{array}$$

where *Y*_wt_ is the mean *Nursing Care Score* in ward w at time t, *β*_0w_ is a random intercept defined by the second equation, *N*_w_ is a dummy variable indicating that ward w is in Trust B, *G*_bw_ is a dummy variable indicating the ward was in the Basic Feedback group, *G*_pw_ is a dummy variable indicating the ward was in the *Feedback Plus* group, *t* is the time in months since the baseline survey was conducted, and *ϵ*_wt_ and *δ*_w_ are random variables with zero mean and standard deviations *σ*_*ϵ*_ and *σ*_*δ*_ respectively. The model implies that there is a fixed effect of Trust (*β*_1_), main effects of treatment group, which allows for there to be different mean *Nursing Care Scores* at baseline observation in the different groups (*β*_2_ and *β*_3_), a main effect of time (*β*_4_), and interaction effects of treatment group and time, which allows us to see whether the direction and rate of change in nursing score varies across treatment groups (*β*_5_ and *β*_6_).

The hypotheses to be tested involve tests of the statistical significance of *β*_5_ and *β*_6_. If *Basic Feedback* and *Feedback Plus* are effective, we would expect that *β*_5_ > 0 and *β*_6_ >0 and if *Feedback Plus* is better than *Basic Feedback*, we would expect *β*_6_ > *β*_5_.

## Results

### Response rates and numbers

Surveys were conducted at six intervals in Trust A, and three in Trust B, where it was curtailed due to administrative difficulties following a change of Director of Nursing. In 18 wards, a total of 9,565 patients were surveyed and 4,236 returned usable questionnaires, representing a response rate of 47% once deceased patients and questionnaires that were returned undelivered had been accounted for.

### Survey contractor’s report

The survey contractor reported that the number and type of calls to the free telephone helpline about the study’s surveys were consistent with their usual experience of carrying out national inpatient surveys. The contractors reported that no new difficulties had arisen as a result of carrying out ward-based surveys. The resources required to implement the ward-level surveys were similar (per patient surveyed) to those needed to conduct the national inpatient surveys.

### Nurses’ reactions to patient feedback

#### *Basic feedback wards*

Printed feedback alone did not seem to stimulate interest in survey results. When asked, none of the *Basic Feedback* ward managers identified specific actions resulting from the printed results, and none of them initiated contact with the researchers.

#### *Feedback plus wards*

The meetings varied in the number of attendees: some were attended only by the ward manager, while others included most of the ward’s nursing staff. Matrons attended some meetings, and they usually had a positive influence: offering suggestions for improvement, encouraging ward nurses to take responsibility for results or supporting efforts to implement changes.

The *Feedback Plus* ward meetings seemed to facilitate nurses’ engagement. Patients’ written comments were particularly useful in illustrating quantitative results and stimulating their interest. However, there remained many challenges to engaging nurses and maintaining their interest. During each meeting, researchers needed to prompt them several times to return their focus to understanding the patient feedback and planning strategies for improvement. Without these prompts, nurses were inclined to discuss other more general matters, such as the many difficulties they experienced in fulfilling their duties; staffing shortages; NHS policy or their perceptions of hospital managers. The meetings were usually interrupted to allow nurses to attend to patients’ immediate needs. Several nurses said that they felt under greater pressure to focus on formally-monitored ward performance criteria, such as incidences of falls, pressure ulcers, clostridium difficile and methicillin-resistant staphylococcus aureus (MRSA), rather than patient survey results, which were not part of formal ward performance measures. Nurses were often reluctant to acknowledge negative feedback and many of them questioned the validity of results or argued that they could not do anything to improve the care they offered. For example:

“Patients think we are talking in front of them as if they are not there because we have to talk quietly to maintain patient confidentiality.”

“Some patients use call buttons for trivial reasons so it would not be a good use of our time to answer them all immediately.”

“Ward/toilet areas are often dirty because our patients are particularly untidy.”

### Actions to improve patient care

After the first meeting on each ward, at subsequent meetings the researchers asked nurses what actions they had taken to improve survey results, but it was difficult to ascertain clear examples of innovations in patient care. Their most common response was that nurse managers had raised the issue at daily handover meetings or in ward meetings and had reminded nurses of the importance of fulfilling their duties relating to ensuring patients’ experiences were positive.

### Changes in *nursing care scores* by ward

Table 1 shows the baseline and final *Nursing Care Scores* for each participating ward.

**Table 1:** **Mean *Nursing Care Scores *by ward at baseline and final surveys**

**Trust**	**Experimental Group**	**Baseline score**	**N**	**Std. Dev.**	**Final score**	**N**	**Std. Dev.**	**Change**
Trust A	*Feedback Plus* Ward 1	80.5	203	15.8	80.1	100	16.4	-0.4
Trust A	*Feedback Plus* Ward 2	82.6	111	14.7	83.7	89	14.9	1.0
Trust A	*Feedback Plus* Ward 3	76.0	59	18.6	76.9	67	16.5	0.8
Trust B	*Feedback Plus* Ward 1	65.9	35	17.0	63.2	54	23.8	-2.7
Trust B	*Feedback Plus* Ward 2	64.3	34	22.6	54.0	23	25.8	-10.4
Trust B	*Feedback Plus* Ward 3	65.2	15	19.7	71.8	11	20.7	6.5
Trust A	*Basic Feedback* Ward 1	74.6	38	19.1	71.7	28	19.8	-2.9
Trust A	*Basic Feedback* Ward 2	71.4	46	20.3	64.9	41	18.9	-6.5
Trust A	*Basic Feedback* Ward 3	83.7	166	16.6	68.2	34	23.3	-15.5
Trust B	*Basic Feedback* Ward 1	75.7	24	14.1	62.6	12	21.1	-13.0
Trust B	*Basic Feedback* Ward 2	76.6	14	20.3	71.7	12	15.2	-4.9
Trust B	*Basic Feedback* Ward 3	71.4	13	27.8	63.1	15	25.1	-8.4
Trust A	Control Ward 1	74.7	72	17.7	66.8	66	21.5	-7.9
Trust A	Control Ward 2	76.2	43	17.2	68.7	43	21.6	-7.5
Trust A	Control Ward 3	81.1	29	13.9	77.0	26	16.6	-4.1
Trust B	Control Ward 1	69.7	31	20.9	72.7	28	16.7	3.0
Trust B	Control Ward 2	78.5	20	11.4	62.2	10	20.9	-16.3
Trust B	Control Ward 3	69.4	34	20.4	72.4	25	17.0	3.0

### Multilevel regression

Estimates of the regression parameters in this model are shown in Table 2. The main effect of Trust (*β*_1_) indicates that the average *Nursing Care Score* of the wards in Trust B was 9.27 lower than that of the wards in Trust A at the time of the baseline assessment. Similarly, the main effects of treatment group (*β*_2_ and *β*_3_) account for differences in the baseline scores of the wards in these groups. These are not statistically significant, as would be expected given the random assignment of wards to treatment group.

**Table 2:** Multilevel regression estimates (standard errors in parentheses)

	
Intercept (*γ*_0_)	78.7 (2.12)*
Trust B (*β*_1_)	-9.27 (1.97)*
Basic Feedback (*β*_2_)	-0.65 (2.62)
Feedback Plus (*β*_3_)	-2.47 (2.62)
Month (*β*_4_)	-0.41 (0.14)*
Basic Feedback x month (*β*_5_)	-0.03 (0.19)
Feedback Plus x month (*β*_6_)	0.46 (0.20)*
Intercept standard deviation (σ_δ_ )	3.58
Residual standard deviation (*σ*_*ϵ*_)	3.82
Log Likelihood	231.3

Test of hypothesis that Basic Feedback x month = Feedback Plus x month: Chi-square statistic = 5.99, 1 degree of freedom, p = 0.014.

Test of hypothesis that Control x month = Feedback Plus x month: Chi-square statistic = 5.34, 1 degree of freedom, p = 0.02.

* p < 0.05.

The main effect of months since the baseline survey (*β*_4_) shows that the average score among the wards involved in the study was declining by 0.41 each month. The two terms that are interactions of group and time show the extent to which the scores change over the period of the study in the different groups. The *Control* group is the excluded category, so the average monthly change in scores in these wards was just the main effect of month discussed above. In the case of the *Basic Feedback* group, the estimate of the interaction effect (*β*_5_) is small and not statistically significant, so we can conclude that scores change in a way that it essentially identical to those of wards in the *Control* group. However, the estimated interaction effect in the *Feedback Plus* group (*β*_6_) is 0.46 and is statistically significant. We can also reject the null hypothesis that *β*_5_ = *β*_6_ (*χ*^2^ = 5.99, p = 0.014). This implies that the *Nursing Care Scores* in the *Feedback Plus* group change at a different rate to those in either the *Control* or *Basic Feedback* groups. The fact that *β*_6_ is positive implies that patients in *Feedback Plus* wards experienced an improvement in scores over the study relative to patients in the other wards. Over 18 months the difference in *Nursing Care Score* between *Control* and *Feedback Plus* wards is 8.28 ± 7.2 (*p* = 0.02).

**Figure 1:**
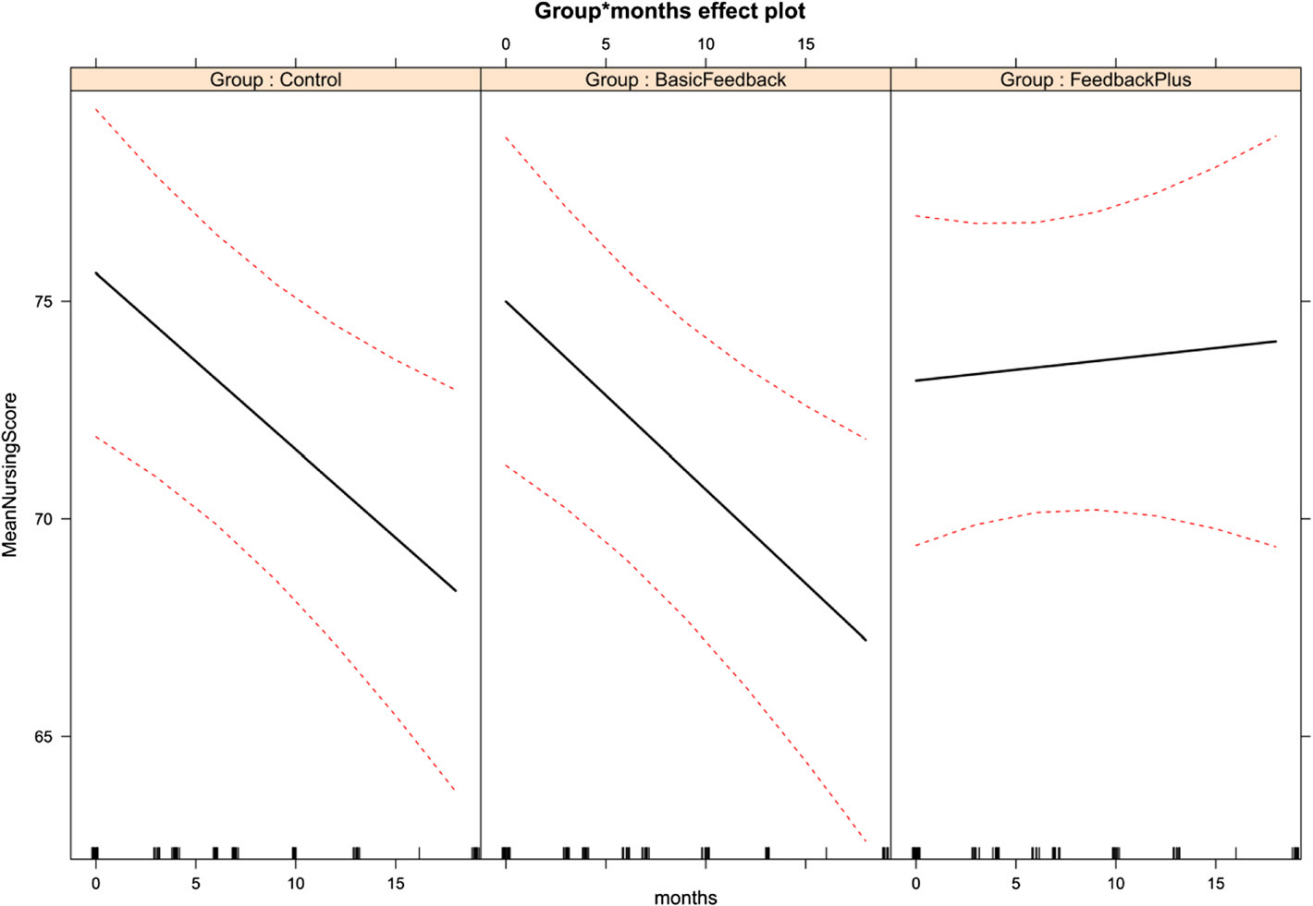
**Estimated change in *Nursing Care Score *over time for the three treatment groups, with 95% confidence intervals.**

The estimated change in *Nursing Care Score* over time for the three treatment groups is shown graphically in Figure 1. We can clearly see the marked declines in scores in the *Control* and *Basic Feedback* wards and the slight increase in *Feedback Plus* wards.

### Sample size calculation

Using the figures in Table 1 applied to the formula by Ukoumunne et al. [28], the average cluster size (*m*) was 45 at each time point. Using the estimates of the standard deviations of the random effects in Table 2 gives an estimate of the intraclass correlation coefficient (*ρ*) of 0.02. The observed standard deviation of the outcome variable (σ) is 6.5, so an effect size of 0.2 would correspond to a change in the outcome measure of 1.3. Taking these as the values required to calculate the sample size, and assuming one-tailed tests, an α level of 0.05 and power of 0.8, gives a sample size of 15 wards in the Feedback Plus group and 15 wards in the Control group. This calculation uses the formula given below:

$$\frac{2\sigma^{2}\left(1-\left(m-1\right)\rho \right){\left(z_{\alpha }+z_{\beta }\right)}^{2}}{md^{2}}$$

## Discussion

### Feasibility

This pilot provides evidence that conducting ward-level surveys, and providing nurses with ward-specific results in printed form by letter and in ward meetings are feasible. Ward managers co-operated by attending ward meetings, and allowing other nursing staff to attend them.

### Effectiveness of the intervention

The structured ward meetings of *Feedback Plus* were effective in encouraging nurses to pay attention to their survey results, facilitating ownership of results and planning changes in practice. The meetings provided vital opportunities for challenging nurses’ scepticism about the relevance of the results, correcting misunderstandings about the survey method and explaining its strengths compared to other less rigorous methods. There is no evidence that the weaker intervention (*Basic Feedback*) led to improved patients’ experiences, or that nurses pay attention to patient survey results when they are only in printed form. Patient survey results in *Feedback Plus* were significantly more improved than in *Basic Feedback* or *Control.*

### Strengths and weaknesses of the study

The randomisation of wards to conditions made it possible to separate the effects of the intervention from extraneous factors in a way that a pre- and post-test within a single setting could not. With more than 4,000 patient responders, this was a fairly large-scale study, but the clustering of patients in wards for the purposes of computing wards’ average scores meant that the number of measurement units was only 18 wards in two hospital trusts, so its generalisability and statistical power were limited.

The qualitative data collected by taking notes in ward meetings and conducting telephone interviews with ward managers provided useful insights into the mechanisms by which patient feedback is understood by nurses. However, the methods by which information about nurses’ reactions to the feedback were collected could have been specified in greater detail in the research design, and given a more formal structure.

The standard 90-question CQC Inpatient Questionnaire was used but it covers a wider range of inpatients’ experiences than was needed for this study. A shorter instrument focusing on ward nursing care would have been more efficient and might have improved the response rate. These findings also suggest that the standard Inpatient Questionnaire comprises more than the optimal number of questions, and clinicians’ focus could improve if a shorter questionnaire were used for the National Inpatient Surveys. The use of a composite *Nursing Care Score*, based on questions that cover a wide range of aspects of nursing care, reduced the risk that any changes detected were isolated examples that masked deteriorations in other aspects of care.

Although the difference in *Nursing Care Scores* in *Feedback Plus* wards compared to both *Basic Feedback* and *Control* wards was statistically significant, the change in scores in the *Feedback Plus* wards was small. Larger improvement may be achieved when there is not the downward trend on scores that we observed in other wards in this study, but this cannot be known without further trials.

### Policy implications

This study offers some explanations for the hitherto low impact of England’s patient survey programme. Elements of *Feedback Plus*: a validated survey instrument; robust survey methods; ward-specific surveys; facilitated ward meetings; the inclusion of patients’ written comments alongside numerical survey results; and the involvement of matrons in communicating and discussing patient feedback have been shown to be effective in engaging clinicians and thereby strengthening the impact of patient surveys.

### Future research

In future studies, a *Basic Feedback* condition would not be necessary, since the findings of this study provide good evidence that it is not sufficient to engage nurses’ interest. This would allow more wards to be allocated to a modified *Feedback Plus* condition and would further increase its power. A more detailed process evaluation plan should be included, along with a clearer structure for gathering information about nurses’ reactions to the feedback.

## Conclusion

This study shows that merely informing nurses of patient survey results in writing is not sufficient to stimulate their interest, even when results are disaggregated at ward level. However, the addition of facilitated feedback in the form of ward meetings can have an impact. To date, few studies have demonstrated that an intervention can improve patients’ experiences. This study provides preliminary evidence that patient feedback can improve patient survey results.

## Abbreviations

NHS: National Health Service; CQC: Care quality commission; MRSA: Methicillin-resistant staphylococcus aureus.

## Competing interests

The authors declare that they have no competing financial interests. Rachel Reeves was employed in the development and management of the national inpatient survey at Picker Institute Europe from 2001 to 2005.

## Authors’ contributions

RR: Original idea for the study; study design; literature search and review; descriptive statistics and preliminary data analysis. Main author of abstract, introduction, methods, results and discussion. EW: Contributed to study design, literature review, data interpretation, reviewed all sections of the manuscript. DB: Designed and conducted multi-level data analysis and wrote sections of results and discussion relating to multi-level analysis. Reviewed all sections of the manuscript. All authors read and approved the final manuscript.

## Pre-publication history

The pre-publication history for this paper can be accessed here:

http://www.biomedcentral.com/1472-6963/13/259/prepub
